# A Two-Stage Kalman Filter-Based Carrier Tracking Loop for Weak GNSS Signals

**DOI:** 10.3390/s19061369

**Published:** 2019-03-19

**Authors:** Yan Cheng, Qing Chang, Hao Wang, Xianxu Li

**Affiliations:** 1 School of Electronic and Information Engineering, Beihang University, Beijing 100191, China; chengyan@buaa.edu.cn (Y.C.); wanghao1989@buaa.edu.cn (H.W.); 2 State Grid Information and Telecommunication Branch, Beijing 100761, China; lixianxu@buaa.edu.cn

**Keywords:** GNSS carrier tracking, high sensitivity, Kalman filter, reduce convergence time

## Abstract

For global navigation satellite system receivers, Kalman filter (KF)-based tracking loops show remarkable advantages in terms of tracking sensitivity and robustness compared with conventional tracking loops. However, to improve the tracking sensitivity further, increasing the coherent integration time is necessary, but it is typically limited by the navigation data bit sign transition. Moreover, for standard KF-based tracking receivers, the KF parameters are initialized by the acquired results. However, especially under weak signal conditions, the acquired results have frequency errors that are too large for KF-based tracking to converge rapidly to a steady state. To solve these problems, a two-stage KF-based tracking architecture is proposed to track weaker signals and achieve faster convergence. In the first stage, coarse tracking refines the acquired results and achieves bit synchronization. Then, in the second stage, fine tracking initializes the KF-based tracking by using the coarse tracking results and extends the coherent integration time without the bit sign transition limitation. This architecture not only utilizes the self-tuning technique of the KF to improve the tracking sensitivity, but also adopts the two-stage to reduce the convergence time of the KF-based tracking. Simulation results demonstrate that the proposed method outperforms conventional tracking techniques in terms of tracking sensitivity. Furthermore, the proposed method is compared with the standard KF-based tracking approach, proving that the proposed method converges more rapidly.

## 1. Introduction

For a global navigation satellite system (GNSS) receiver, accurate navigation and timing services are readily available in open outdoor environments. However, when GNSS signals pass through challenging environments, such as dense foliage, urban canyons, and indoor environments, GNSS signals are attenuated substantially. Conventional GNSS receivers have difficulty processing such weak signals. As one of the most important parts of a GNSS receiver, the carrier tracking loop is extremely fragile under weak signal conditions, and its threshold limits the unaided receiver performance [1].

Various efforts have been devoted to improving the robustness of the carrier tracking loop under weak signal conditions. The conventional phase-locked loop (PLL) has been widely used in the carrier tracking loop. The typical strategy for improving the PLL sensitivity is to extend the integration time. Unfortunately, extending the integration time requires narrower filter bandwidths, which conflict with the dynamic performance of the PLL. The frequency-locked loop (FLL) is also commonly used in the tracking loop, and it can tolerate large, sustained frequency errors [2]. In [3], a FLL-assisted-PLL tracking loop was designed, which can resolve the conflict between the dynamic performance and the accuracy performance. However, Ref. [4] also demonstrated that the conventional tracking loop assumes that the input GNSS signal is deterministic. Consequently, the conventional loop is difficult to keep well locked in time-varying environments.

The Kalman filter (KF) is a time-variant system, and it can be substituted for the conventional PLL. According to the literature, KF-based tracking shows superior performance in terms of tracking sensitivity. In [5], the KF-based tracking loop was employed to track weak GNSS signals. In addition, Refs. [6,7] both demonstrated that KF-based tracking can track weaker signals than traditional tracking approaches. To improve tracking sensitivity, KF-based tracking also requires an increased integration time, which has been illustrated in [8]; however, the integration time should be chosen optimally to compromise between the sensitivity and the loop update rate. Hence, Ref. [9] proposed an adaptive PLL, which could adaptively adjust the integration time to track weak and dynamic signals. Moreover, Ref. [10] assumed that the data bit sign had been eliminated before employing KF-based tracking because data bit reversals could cause incorrect KF estimations. Accordingly, it is necessary to achieve bit synchronization before KF-based tracking, especially when the coherent integration time should be extended for tracking weaker signals.

The conventional tracking usually achieves bit synchronization before entering the tracking stage. For instance, in [11], it was assumed that bit synchronization should be employed initially to extend the integration time in conventional tracking. Similarly, Ref. [12] proposed the control strategy in conventional tracking that achieved the bit synchronization in the first tracking phase. However, achieving bit synchronization before KF-based tracking has not yet been investigated.

Additionally, the main drawback of KFs is the need of the exact prior parameters to initialize KF values. For standard KF-based tracking receivers, the KF-based tracking parameters are typically initialized according to the acquired results, which was presented in [13,14]. However, because of the large frequency error of the acquisition results under weak signal conditions, it is difficult for the KF algorithm to converge rapidly. KF-based tracking requires an accurate initialization to keep lock under weak signal conditions. In [15], it was also demonstrated that accurate initial values can decrease the possibility of false frequency-locking in weak signal tracking. In [5], fine acquisition results were used to initialize the KF-based tracking. However, the fine acquisition stage could not achieve bit synchronization and it needed an additional Bayesian approach to determine bit signs.

To overcome these problems, a two-stage KF-based tracking loop is proposed. This scheme involves both coarse tracking and fine tracking. The coarse tracking stage refines the acquisition results and reduces the large frequency errors, while the fine tracking stage adopts the KF-based PLL to track. An important part between the coarse tracking and the fine tracking is the judgment of the bit synchronization. If the bit synchronization is not successful, the receiver will not enter the fine tracking stage and coarse tracking will continue. In this way, the data bit signs are known in the fine tracking stage. Hence, the coherent integration time can be increased in the fine tracking stage to track weaker signals. In addition, the proposed method can use the coarse tracking results to initialize the KF parameters. Accordingly, the proposed tracking algorithm not only implements KF-based tracking to improve the tracking sensitivity, but also employs two-stage tracking to converge rapidly.

The remainder of this paper is organized as follows. In the Section 2, the GNSS signal model is presented. Then, the Section 3 describes the conventional two-stage tracking method. In the Section 4, the proposed two-stage KF-based tracking architecture is described in detail. In the Section 5, simulations are performed to evaluate the tracking sensitivity, convergence speed, and weak dynamic signal performance. Finally, the conclusions are presented in the Section 6.

## 2. Signal Model

The GNSS radio frequency (RF) signal is received by a user antenna. After down-conversion and analog-to-digital conversion (ADC), the RF signal is converted into the discrete intermediate frequency (IF) signal. The digital IF signal can be modeled as [10]:

(1) $$r_{IF}(nt_{s})=A\cdot D(nt_{s}-\tau )\cdot c(nt_{s}-\tau )\cdot \cos(2\pi (f_{IF}+f_{d})nt_{s}+\varphi_{0})+w(nt_{s})$$

where $t_{s}$ is the sampling duration, *A* is the IF signal amplitude, $D(nt_{s})$ denotes the navigation data bit, $c(nt_{s})$ is the pseudo-random number (PRN) spreading code, τ is the code delay of the received signal, $f_{IF}$ denotes the intermediate frequency in hertz, $f_{d}$ represents the carrier Doppler shift in hertz, $\varphi_{0}$ is the initial carrier phase in radians, and $w(nt_{s})$ represents the additive zero-mean white Gaussian noise (AWGN).

In each tracking channel of the receiver, the IF signal is multiplied with two local carriers that are in-phase and 90° shifted, to wipe off the carrier frequency. Then, the result is multiplied by the local code. Finally, its output is passed to the integration and dump blocks to generate *I* and *Q* correlated signals. The prompt branch signals, *I* and *Q*, can be described as [4]:

(2) $$I(k)=A\cdot N(k)\cdot R{\left(\tau -\hat{\tau }\right)}_{k}\cdot \frac{\sin(\pi \cdot \delta f(k)\cdot T_{s})}{\pi \cdot \delta f(k)\cdot T_{s}}\cdot \cos({\bar{\delta \varphi }}_{k})+w_{I}(k)$$

(3) $$Q(k)=A\cdot N(k)\cdot R{\left(\tau -\hat{\tau }\right)}_{k}\cdot \frac{\sin(\pi \cdot \delta f(k)\cdot T_{s})}{\pi \cdot \delta f(k)\cdot T_{s}}\cdot \sin({\bar{\delta \varphi }}_{k})+w_{Q}(k)$$

where k is the updated index of the correlated values, A denotes the signal amplitude, N(k) is the number of samples accumulated from the correlator, $R{\left(\tau -\hat{\tau }\right)}_{k}$ represents the autocorrelation function of the PRN code, $\hat{\tau }$ denotes the code delay of the local prompt PRN code, δf(k) is the carrier frequency error in hertz, $T_{s}$ is the coherent integration time (CIT) in seconds, ${\bar{\delta \varphi }}_{k}$ is the average carrier phase error over $T_{s}$, and $w_{I}(k)$ and $w_{Q}(k)$ are both white Gaussian noise that are uncorrelated and have the same power.

## 3. Conventional Two-Stage Carrier Tracking Strategy

Carrier estimation consists of carrier acquisition and carrier tracking. Carrier acquisition is a search process, and the frequency search step is typically set to 666.67 Hz. Therefore, the maximum frequency error of the acquisition results is 333.34 Hz. Due to the large frequency error, it takes a long time to track the signal. To decrease the tracking time and achieve fast convergence, it is necessary to design a two-stage carrier tracking control strategy, which was presented in [12].

**Figure 1:**
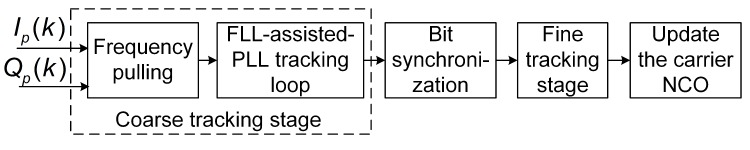
Block diagram of the conventional two-stage carrier tracking control strategy.

Figure 1 shows the block diagram of the conventional two-stage carrier tracking strategy. This architecture contains both coarse tracking and fine tracking. Hereafter, each part of the architecture is presented in detail.

### 3.1. Coarse Tracking Design

Figure 1 presents that coarse tracking stage, which contains two parts. They are the frequency pulling module and the FLL-assisted-PLL tracking loop, which are introduced in the following subsections.

#### 3.1.1. Frequency Pulling Algorithm

**Figure 2:**
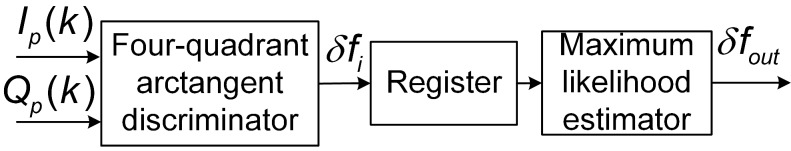
Block diagram of the frequency pulling algorithm.

The inputs of the frequency pulling module are the prompt branch of the orthonormal correlator outputs, I(k) and Q(k), as expressed in (2) and (3). The block diagram of the frequency pulling model is shown in Figure 2.

The formula for the four-quadrant arctangent discriminator is given by [1]:

(4) $$\{\begin{array}{c} cross=I_{p}(k-1)Q_{p}(k)-Q_{p}(k-1)I_{p}(k)\\ dot=I_{p}(k-1)I_{p}(k)+Q_{p}(k-1)Q_{p}(k) \end{array}$$

where $I_{P}(k)$ and $Q_{P}(k)$ are the correlation values of the prompt branch at time k. The phase error and frequency error are expressed by [1]:

(5) $$\delta \varphi =\mathrm{ATAN}2(cross,dot)=2\pi T_{s}\delta f(k)$$

(6) $$\delta f_{i}=\delta f(k)=\frac{\delta \varphi }{2\pi T_{s}}$$

where ATAN2(x,y) is the four-quadrant arctangent function, which returns the result in radians. The unit of $\delta f_{i}$ is hertz.

Due to the randomness of the receiver dynamic, the estimated value, $\delta f_{i}$, can be regarded as a random variable. The output of the frequency pulling model, $\delta f_{out}$, is computed by the maximum likelihood estimator of all observation measurements, $\delta f_{i}$. The outlier elimination method is adopted as the maximum likelihood estimator. The calculation method is as follows:

(7) $$\delta f_{out}=\frac{1}{N-2}\left[\left(\sum_{i=1}^{N}\delta f_{i}\right)-\delta f_{\max}-\delta f_{\min}\right]$$

where N is the total number of observations during the frequency pulling algorithm, and $\delta f_{\max}$ and $\delta f_{\min}$ are the maximum and minimum, respectively, of the N observation values.

#### 3.1.2. FLL-Assisted-PLL Tracking Loop Design

**Figure 3:**
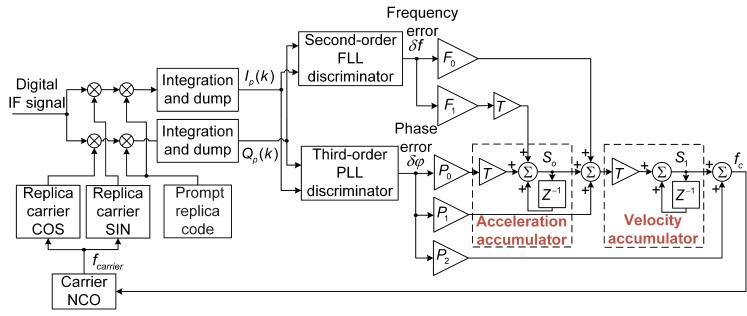
Block diagram of the second-order FLL assisting third-order PLL tracking loop. (NCO: numerically controlled oscillator; FLL: frequency-locked loop; PLL: phase-locked loop).

To track the dynamic signals, the FLL-assisted-PLL tracking loop is selected. Figure 3 shows the block diagram of the second-order FLL assisting the third-order PLL tracking loop, as described in [1].

In Figure 3, the updated tracking loop can be calculated as:

(8) $$\left\{ \begin{array}{l} S_{0}=S_{0}+(\delta \varphi \cdot P_{0}+\delta f\cdot F_{1})\cdot T\\ S_{1}=S_{1}+(\delta \varphi \cdot P_{1}+S_{0}+\delta f\cdot F_{0})\cdot T\\ f_{c}=S_{1}+\delta \varphi \cdot P_{2}\\ f_{carrier}=f_{ref}+f_{c} \end{array}\right.$$

where δφ and δf are the outputs of the PLL and FLL discriminators, respectively; $F_{0}$, $F_{1}$, $P_{0}$, $P_{1}$, and $P_{2}$ are the coefficients of the FLL and PLL tracking loops; T is the updated period of the tracking loop, which is typically the same as the predetection integration time; $f_{c}$ is the carrier frequency error after the update; and $f_{carrier}$ is the local carrier frequency after adjustment. The discriminator results, δφ and δf, are computed as described in Table 1.

### 3.2. Fine Tracking Design

When the dynamic stress of the received IF signal is large, coarse tracking can substantially weaken the dynamic stress. The FLL-assisted-PLL tracking loop outperforms the PLL tracking loop under dynamic signal conditions, however, the measurement accuracy of the FLL is worse than that of the PLL. Hence, the fine tracking stage should be employed to improve the tracking accuracy further.

As shown in Figure 1, the switch between coarse tracking and fine tracking depends on whether the bit synchronization is successful. Once bit synchronization is achieved, the receiver enters the fine tracking stage. 

For fine tracking, a third-order PLL is utilized to track the carrier phase accurately. The estimated carrier phase error between the locally generated signal and the received signal in the fine tracking can be obtained by the two-quadrant arctangent discriminator.

Owing to the success of the bit synchronization, the limitation of the data bit sign has been eliminated. CIT can be increased to track weak signals. Nonetheless, the limitation, $B_{n}\cdot T\ll 0.5$, should also be considered. Therefore, the design of the CIT should balance the sensitivity and the normalized loop bandwidth, namely, $B_{n}\cdot T$, as presented in [16].

## 4. KF-Based Two-Stage Carrier Tracking Loop

The conventional two-stage tracking architecture is not robust under harsh environments, especially under weak signal conditions. To improve the tracking sensitivity and achieve fast convergence, a KF-based coarse-to-fine carrier tracking strategy is proposed. The proposed two-stage KF-based carrier tracking loop is analyzed in this section.

### 4.1. Basic Equations of KF-Based Tracking

A linear KF state space system model can be described as [17]:

(9) $$x_{k+1}=\Phi x_{k}+n_{k}$$

(10) $$z_{k+1}=Hx_{k}+v_{k}$$

Equation (9) is the discrete state equation, where $x_{k}$ is the state vector at the $k^{th}$ epoch, Φ is the state transition matrix, and $n_{k}$ represents the process noise matrix. Equation (10) is the discrete measurement equation, where $z_{k+1}$ is the measurement vector at the ${(k+1)}^{th}$ epoch, H is the measurement matrix, and $v_{k}$ represents the measurement noise vector.

For the third-order KF-based carrier tracking loop, $x_{k}={\left[\begin{array}{ccc} \Delta \varphi_{k} & \omega_{k} & \alpha_{k} \end{array}\right]}^{T}$ and the discrete state equation and measurement equation are expressed as:

(11) $$\left[\begin{array}{c} \Delta \varphi_{k+1}\\ \omega_{k+1}\\ \alpha_{k+1} \end{array}\right]=\left[\begin{array}{ccc} 1 & T & T^{2}/2\\ 0 & 1 & T\\ 0 & 0 & 1 \end{array}\right]\left[\begin{array}{c} \Delta \varphi_{k}\\ \omega_{k}\\ \alpha_{k} \end{array}\right]+n_{k}$$

(12) $$\delta \varphi_{k+1}=\left[\begin{array}{ccc} 1 & T/2 & T^{2}/6 \end{array}\right]\left[\begin{array}{c} \Delta \varphi_{k}\\ \omega_{k}\\ \alpha_{k} \end{array}\right]+v_{k}$$

where $\Delta \varphi_{k}$ represents the carrier phase error in radians, $\omega_{k}$ represents the carrier Doppler shift in rad/s, $\alpha_{k}$ is the carrier Doppler rate in $\mathrm{rad}/s^{2}$, and T is the updated period of the carrier tracking loop in seconds. In (12), $\delta \varphi_{k+1}$ is the measurement vector, which is obtained via a two-quadrant arctangent discriminator. 

For (11), the covariance matrix, Q, of the system noise matrix, $n_{k}$, is [8]:

(13) $$\begin{array}{ll} Q & ={\left(\frac{\omega_{rf}}{C}\right)}^{2}q_{a}\left[\begin{array}{ccc} T^{5}/20 & T^{4}/8 & T^{3}/6\\ T^{4}/8 & T^{3}/3 & T^{2}/2\\ T^{3}/6 & T^{2}/2 & T \end{array}\right]\\ & +{\omega_{rf}}^{2}q_{d}\left[\begin{array}{ccc} T^{3}/3 & T^{2}/2 & 0\\ T^{2}/2 & T & 0\\ 0 & 0 & 0 \end{array}\right]+{\omega_{rf}}^{2}q_{b}\left[\begin{array}{ccc} T & 0 & 0\\ 0 & 0 & 0\\ 0 & 0 & 0 \end{array}\right] \end{array}$$

where $\omega_{rf}$ denotes the nominal carrier frequency in rad/s, $q_{a}$ represents the power spectral density of the random walk process in units of $\left(m^{2}/s^{6}\right)/\mathrm{Hz}$, C denotes the velocity of light, and $q_{d}$ and $q_{b}$ represent the power spectral density of the carrier frequency noise and carrier phase noise, respectively. $q_{a}$ is determined by the line-of-sight (LOS) jerk. If the relative motion between the satellite and the receiver is static, $q_{a}=0\left(m^{2}/s^{6}\right)/\mathrm{Hz}$. $q_{d}$ and $q_{b}$ are caused by local oscillator noise. If the receiver oscillator parameters are known, the values of $q_{d}$ and $q_{b}$ can be calculated by [18]:

(14) $$q_{d}=2\pi^{2}h_{-2}$$

(15) $$q_{b}=\frac{h_{0}}{2}$$

where $h_{0}$ and $h_{-2}$ are the receiver oscillator *h*-parameters. The values of the *h*-parameters are determined by the type of oscillator. Typical values for commonly used oscillators can be found in [18].

In (12), the measurement noise, $v_{k}$, is white Gaussian noise with a variance, $\sigma_{v}^{2}$. The measurement noise covariance, R, for the output of the two-quadrant arctangent discriminator, can be expressed as [16]:

(16) $$R=\sigma_{v}^{2}=E\left[v_{k}{v_{k}}^{T}\right]=\frac{1}{2Tc/n_{0}}\left(1+\frac{1}{2Tc/n_{0}}\right)$$

where $c/n_{0}$ is the carrier-to-noise ratio, which is typically obtained by:

(17) $$c/n_{0}=10^{\frac{C/N_{0}}{10}}$$

The unit of $C/N_{0}$ is dB-Hz.

### 4.2. KF-Based Coarse-to-Fine Carrier Tracking Algorithm

The KF-based tracking loop uses the estimated phase and frequency to predict the estimated values at the next epoch. If the navigation bit sign is flipped, the 180-degree phase ambiguity will occur. In this situation, the KF-based tracking loop will estimate the results incorrectly during the iteration, and the KF-based tracking loop may eventually lose lock. Accordingly, the KF-based tracking method should be used after the data bit signs are known. As demonstrated above, the coarse tracking algorithm can achieve bit synchronization. If bit synchronization is not successful, fine tracking will not be performed. In this way, bit signs in the fine tracking have been well estimated already.

**Figure 4:**
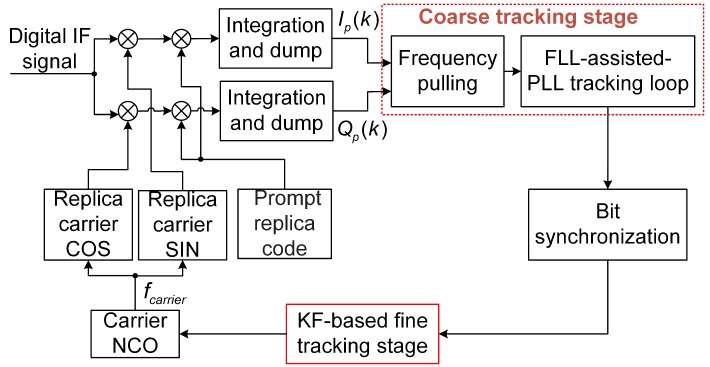
Block diagram of the KF-based coarse-to-fine carrier tracking strategy. (KF: Kalman filter).

Figure 4 depicts the block diagram of the KF-based coarse-to-fine carrier tracking architecture. The in-phase and quadrature integration results of the prompt branch are sent to the coarse tracking loop. The coarse tracking stage reduces the frequency error to achieve bit synchronization. The main function of the frequency pulling algorithm is to reduce the acquired frequency error to the working range of the FLL-assisted-PLL tracking loop, as shown in Figure 2. The FLL-assisted-PLL tracking loop obtains a rough estimate of the carrier Doppler frequency and phase to achieve bit synchronization. Then, if bit synchronization is successfully achieved, the fine tracking stage will employ the KF algorithm to accurately estimate the phase and Doppler frequency. Finally, the output of the KF-based fine tracking is used to update the frequency and phase of the carrier NCO.

The steps of the algorithm are detailed as follows:

(1) Initialization. Initialize the bandwidths and coefficients of the PLL and FLL for the coarse tracking stage. Set $B_{nPLL}$, $B_{nFLL}$, and the coefficients of the second-order FLL and third-order PLL for the FLL-assisted-PLL tracking loop.
(2) The first step of the coarse tracking: Frequency pulling. First, set the integration time, $T_{\mathrm{pull}}$ = 1 ms, to ensure fast convergence to the steady state and read out the correlator outputs of the prompt branch. Then, calculate the phase error, δφ, from the four-quadrant arctangent discriminator every 1 ms. To eliminate the outliers of the frequency error, $\delta f_{i}$, save 20 frequency errors and use the maximum likelihood estimator in (7) to obtain the frequency residual, $\delta f_{out}$. Finally, use the frequency error to update the carrier NCO.
(3) The second step of the coarse tracking: The FLL-assisted-PLL tracking loop. When the frequency pulling algorithm has finished, the tracking stage switches to the FLL-assisted-PLL tracking loop. First, in the second-order FLL, the integration time, T, is set to $T_{\mathrm{FPLL}}$. Next, the cross-product discriminator is used to obtain the frequency error, δf, every $2\cdot T_{\mathrm{FPLL}}\mathrm{ms}$. Then, in the third-order PLL, the two-quadrant arctangent discriminator is utilized to obtain the phase error, δφ, every $T_{\mathrm{FPLL}}\mathrm{ms}$. Finally, update the tracking loop via (8).
(4) Bit synchronization. If t > 1000 ms, bit synchronization is employed. t denotes the number of times that the tracking procedure enters the coarse tracking stage and this typically occurs every 1 ms. 1000 ms represents that 1 bit navigation data has entered the coarse tracking stage. Only when t > 1000 ms can the bit synchronization be employed. Once bit synchronization succeeds, the KF-based fine tracking will be implemented; otherwise, coarse tracking will continue.
(5) KF-based fine tracking. After successful bit synchronization, the effect of the navigation data bit transition is wiped off; hence, the integration time can be extended to 20 ms. First, read out the correlator outputs of the prompt branch and initialize the KF parameters: The process noise covariance matrix, Q; the measurement noise covariance matrix, R; the state vector, $x_{k}$; and the state error covariance matrix, $P_{0}^{-}$. According to the previous section, the initiation of Q depends on (13). The updated period of the KF-based loop, T, can be designed according to the $C/N_{0}$ estimate. If the estimated $C/N_{0}$ is low, the updated period, T, can be increased to track weak signals; otherwise, T cannot be set too long. The initialization of R depends on (16). The initialization of $x_{k}$ and $P_{0}^{-}$ depends on the coarse tracking results. Then, the two-quadrant arctangent discriminator is performed to obtain the phase errors every Tms, which are used to provide the measurement values, $z_{k}$. Finally, use KF recursive equations iteratively to update the carrier phase error and the Doppler frequency. The obtained phase error and Doppler frequency are used to update the carrier NCO.

In contrast to the standard KF-based tracking method, the KF initialization in the proposed strategy depends not on the acquired results, but on the coarse tracking results. The coarse tracking results are the FLL-assisted-PLL tracking loop results as shown in Figure 3. The two boxes in Figure 3 that are labeled with $z^{-1}$ from left to right represent the acceleration accumulator and the velocity accumulator, respectively. The prior state vector, namely, ${\hat{x}}_{0}={\left[\begin{array}{ccc} \Delta {\hat{\varphi }}_{0} & {\hat{\omega }}_{0} & {\hat{\alpha }}_{0} \end{array}\right]}^{T}$, can be initialized as follows:

(18) $${\hat{x}}_{0}=\left[\begin{array}{c} \Delta {\hat{\varphi }}_{0}\\ {\hat{\omega }}_{0}\\ {\hat{\alpha }}_{0} \end{array}\right]=\left[\begin{array}{c} 0\\ (\delta \varphi \times P_{1}+\delta f\times F_{0}+S_{0})\times T+S_{1}\\ (\delta \varphi \times P_{0}+\delta f\times F_{1})\times T+S_{0} \end{array}\right]$$

where $\Delta {\hat{\varphi }}_{0}$, ${\hat{\omega }}_{0}$, and ${\hat{\alpha }}_{0}$ are the initial phase error, the initial carrier Doppler frequency, and the carrier Doppler frequency rate, respectively. The other variables are defined as in (8). Because the phase error result of the coarse tracking is less than 1, the initial phase error is set to 0. As shown in Figure 3, ${\hat{\omega }}_{0}$ is set to the velocity accumulator result and ${\hat{\alpha }}_{0}$ is set to the acceleration accumulator result.

The maximum frequency error of the coarse tracking results is substantially less than that of the acquired results. Therefore, the convergence time of the KF-based fine tracking will be reduced. Furthermore, if the initial value of the Doppler frequency is more accurate, the possibility of a false frequency-lock in the KF-based fine tracking loop will be greatly reduced, especially under weak signal conditions.

## 5. Performance Evaluation

To evaluate and validate the proposed algorithm, the tracking algorithms discussed above are implemented on a software Global Positioning System (GPS) receiver that is based on a Visual C++ platform. The receiver directly processes the IF signal, which is read from a data file.

### 5.1. Static Weak Signal Environment Simulation

In this section, the tracking sensitivity and convergence speed of the proposed method are evaluated. Under the static weak signal conditions, nine channels are simulated in this software receiver. The signal simulator emulates the controlled attenuated GPS signal as weak signals.

#### 5.1.1. Sensitivity Test and Analysis

For the nine channels, the $C/N_{0}$ values of the input signals are all set to 45 dB-Hz for the first minute. Then, the signal power is decreased by 2 dB every minute until the $C/N_{0}$ reaches 15 dB-Hz. Thus, the total simulation time of this signal is 960 s. Moreover, the frequency of the simulated IF signal is 1.42 MHz, the sampling rate is 10 MHz, and the signal resolution is 4-bit.

In the simulation, $C/N_{0}$ is estimated via the power ratio method, as presented in [19], using wideband and narrowband powers from the sums of the I and Q samples. Hence, bit synchronization is indispensable for $C/N_{0}$ estimation. If a navigation bit transition appears during the computation of the sums, incorrect power estimation could occur. For the two tracking methods discussed above, bit synchronization was achieved before the fine tracking. Therefore, the power ratio method can be employed in the fine tracking stage. In this experiment, $C/N_{0}$ is estimated every 5 s.

For comparison, four algorithms are evaluated for static weak signals. The parameters of the four tracking algorithms are listed in Table 2. The algorithms, 2-stage conv1 and 2-stage conv2, have conventional two-stage carrier tracking architectures with different fixed bandwidths and CITs in both coarse tracking and fine tracking. The algorithms, 2-stage KF1 and 2-stage KF2, are the proposed KF-based two-stage carrier tracking architectures with different PLL bandwidths and CITs.

For 2-stage KF1 and 2-stage KF2, the initial parameters in the fine tracking should be set first. The initialization of Q and R depends on (13) and (16), where $q_{a}=0\left(m^{2}/s^{6}\right)/\mathrm{Hz}$ and $c/n_{0}=10^{\frac{45}{10}}$. The state error covariance, $P_{0}^{-}$, is initialized by the coarse tracking results. $\sqrt{P_{0}^{-}(2,2)}$ denotes the initial Doppler frequency uncertainty, which was illustrated in [13]. Because the uncertainty of the coarse tracking Doppler results is approximately 100 Hz, $\sqrt{P_{0}^{-}(2,2)}$ is set to 2π·500 with a sufficient margin. Because the phase error result of the coarse tracking is less than 1 cycle, $\sqrt{P_{0}^{-}(1,1)}$ is set to 2π·1. Due to the static signal, $\sqrt{P_{0}^{-}(3,3)}$ is set to 0. Accordingly:

(19) $$P_{0}^{-}=\mathrm{diag}[(2\pi {)}^{2}(2\pi \cdot 500{)}^{2}0]$$

The initial state vector, ${\hat{x}}_{0}={\left[\begin{array}{ccc} \Delta {\hat{\varphi }}_{0} & {\hat{\omega }}_{0} & {\hat{\alpha }}_{0} \end{array}\right]}^{T}$, represents the estimated values from the coarse tracking results. Its initialization depends on Equation (18).

**Figure 5:**
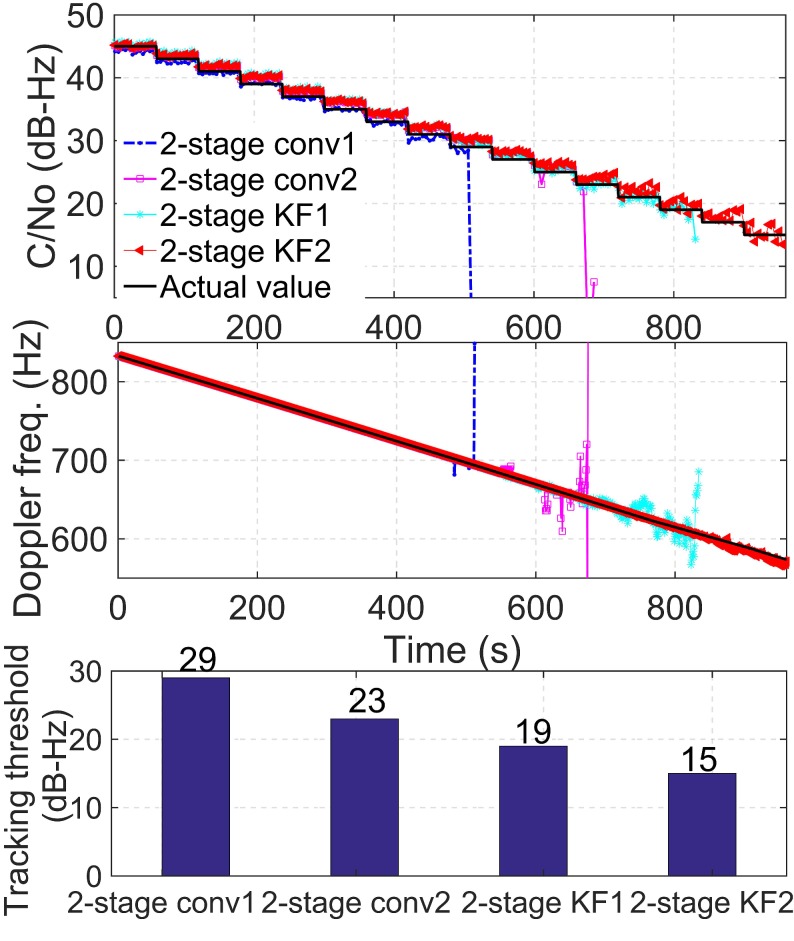
Tracking sensitivity, Doppler frequency, and tracking thresholds estimated by the four tracking algorithms for PRN 14 satellite signals under static weak signal environments. (PRN: pseudo-random number).

For simplicity, only the performance of PRN 14 is illustrated here. Figure 5 shows the sensitivity of the four tracking algorithms. According to the top panel, the $C/N_{0}$ estimates of the four tracking algorithms follow the actual values when $C/N_{0}$ is high. As $C/N_{0}$ decreases, the mismatch between the estimated and actual $C/N_{0}$ becomes increasingly large. The middle panel shows the Doppler frequency that is obtained via the four tracking algorithms. For the static receiver, the Doppler rate is relatively low, which is due to the clock drift of the receiver and the satellite motion. In the bottom panel, the tracking thresholds of the four tracking algorithms are presented.

Comparing these results, the algorithms, 2-stage KF1 and 2-stage KF2, perform better for low $C/N_{0}$ and lose the lock at approximately 19 dB-Hz and 15 dB-Hz, respectively. In contrast, the algorithms, 2-stage conv1 and 2-stage conv2, perform worse and lose the lock at about 29 dB-Hz and 23 dB-Hz, respectively. The algorithm, 2-stage KF1, improves the tracking threshold by approximately 10 dB relative to 2-stage conv1 under the same bandwidth and CIT. Similarly, 2-stage KF2 improves the tracking threshold by approximately 8 dB compared with 2-stage conv2. These results demonstrate that the proposed KF-based two-stage tracking methods outperform the conventional two-stage tracking methods due to the adaptively KF gains in the fine tracking stage when the signal strength decreases.

Comparing the 2-stage conv1 and 2-stage conv2 results, 2-stage conv2 can track the signal until approximately 680 s, when the signal, $C/N_{0}$, reaches the 23 dB-Hz level, while 2-stage conv1 can track the signal at 29 dB-Hz. The tracking threshold of 2-stage conv2 is improved by approximately 6 dB, which is mostly because 2-stage conv2 has a longer CIT and a narrower PLL bandwidth in both the coarse and the fine tracking stages than 2-stage conv1. The narrower bandwidth introduces less noise, and the longer CIT can help track weaker signals.

Compared with 2-stage KF1 and 2-stage KF2, the threshold of 2-stage KF2 is improved by only 4 dB. The reason for this threshold improvement is also the longer CIT and narrower bandwidth. However, the sensitivity improvement in the proposed algorithm is not as noticeable as the conventional algorithm with a 6 dB improvement. Although the coarse tracking stage utilizes the traditional FLL-assisted-PLL tracking loop, the sensitivity of the tracking loop mainly depends on the fine tracking stage once bit synchronization has been achieved. Therefore, when $C/N_{0}$ decreases to a low level, the sensitivity of the proposed tracking loop mainly depends on the time-varying Kalman gains in the fine tracking stage.

In summary, under the same bandwidths and CITs, the proposed method exhibits a substantial performance advantage over the conventional method in terms of the tracking sensitivity. Comparing the 2-stage KF1 to 2-stage conv2, it shows that even with a shorter integration time, the sensitivity of the proposed method is superior to that of the conventional approach. Furthermore, the longer CIT and narrower bandwidth can also improve the sensitivity of the proposed method.

#### 5.1.2. Convergence Speed Test and Analysis

**Figure 6:**
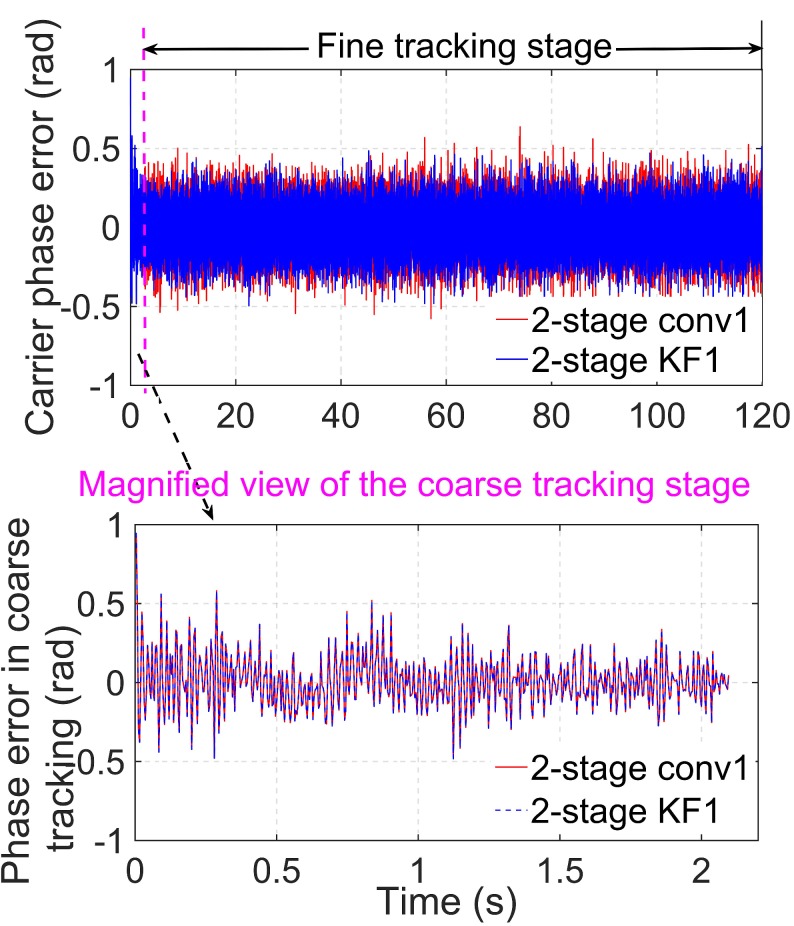
Carrier phase error of the algorithms, 2-stage conv1 and 2-stage KF1, when $C/N_{0}$ = 39 dB-Hz.

Figure 6 shows the carrier phase tracking error when $C/N_{0}$ = 39 dB-Hz and the simulation time is 120 s with the algorithms, 2-stage conv1 and 2-stage KF1, that are described in Table 2. The top panel of the figure is the carrier phase tracking error for the whole tracking stage. The magenta dotted line divides the whole tracking stage into the coarse tracking stage and the fine tracking stage. The bottom panel presents the carrier phase error in detail in the coarse tracking stage. In this simulation, the coarse tracking stage lasts for approximately 2 s, and the remaining time is the fine tracking stage.

In the top panel of Figure 6, the carrier phase errors of the two algorithms are both close to zero, which proves that the two algorithms can track the carrier phase parameters properly. For the 2-stage KF1 algorithm, the seamless connection of the phase error results between the coarse tracking and the fine tracking demonstrates the compatibility between the two tracking stages in the proposed method. In the bottom panel of Figure 6, the carrier phase error results of the two algorithms are the same. This is because the same algorithm is used in the coarse tracking stage.

According to Figure 6, in the first-stage tracking, the carrier phase errors of the two methods both have converged into the steady state. Therefore, there is no need for comparison between the proposed method and the conventional 2-stage method in terms of the convergence speed.

To evaluate the convergence speed, the proposed method is compared with the standard KF-based PLL tracking approach, which contains only one stage. The $C/N_{0}$ value of the simulated signal is set to 39 dB-Hz, and the simulation time is 120 s. For comparison, the standard KF-based PLL with a CIT of 4 ms is compared with the algorithm 2-stage KF1, which is described in Table 2. For the two algorithms, the settings of Q and R are the same according to (13) and (16), where $q_{a}=0\left(m^{2}/s^{6}\right)/\mathrm{Hz}$ and $c/n_{0}=10^{\frac{39}{10}}$. The state error covariance, $P_{0}^{-}$, in the standard KF method is set according to the acquisition results.

**Figure 7:**
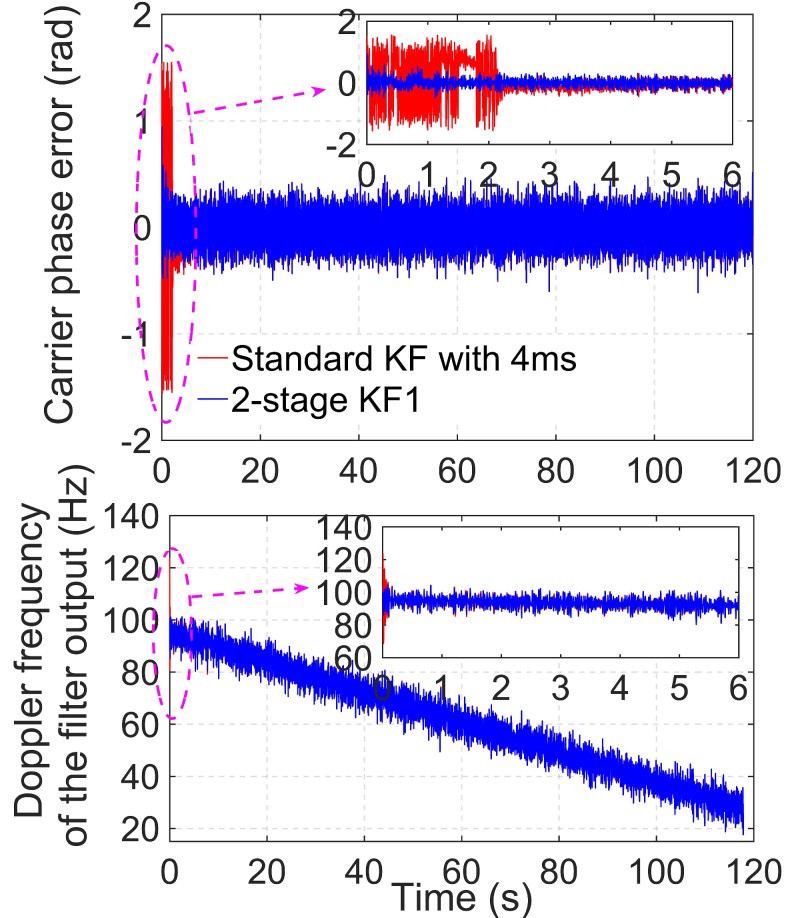
Carrier phase error and Doppler frequency estimations for PRN 9 satellite signals of two KF-based PLL tracking methods under a static signal of $C/N_{0}$ = 39 dB-Hz. (KF: Kalman filter).

Figure 7 shows the carrier phase errors and Doppler frequency estimations of the two different KF-based PLL tracking methods. The top and bottom panels of the figure show the carrier phase errors and the Doppler frequency estimated by the two KF-based tracking approaches, respectively. The small boxes in the top and bottom panels are magnified views of the first 6 s. Evidently, the proposed method converges faster than the standard KF tracking approach. 

The noise of the estimated Doppler frequency in the bottom panel of Figure 7 is larger than that in the middle panel of Figure 5. This is because the Doppler frequency extracted from the filter output contains more noise than that extracted from the velocity accumulator and this was illustrated in detail in [4].

The faster convergence of the proposed method is attributable to the coarse tracking stage that refines the acquisition results. The initial state vectors and the state error covariance of the KF-based fine tracking rely on the coarse tracking results. However, the standard KF-based tracking method uses the acquisition results to initialize the state vector, but the acquisition results are not as accurate as the coarse tracking results of the proposed method. Accordingly, the proposed two-stage KF-based tracking method can achieve faster convergence than the standard KF-based tracking loop.

### 5.2. Dynamic Weak Signal Environment Simulation

**Figure 8:**
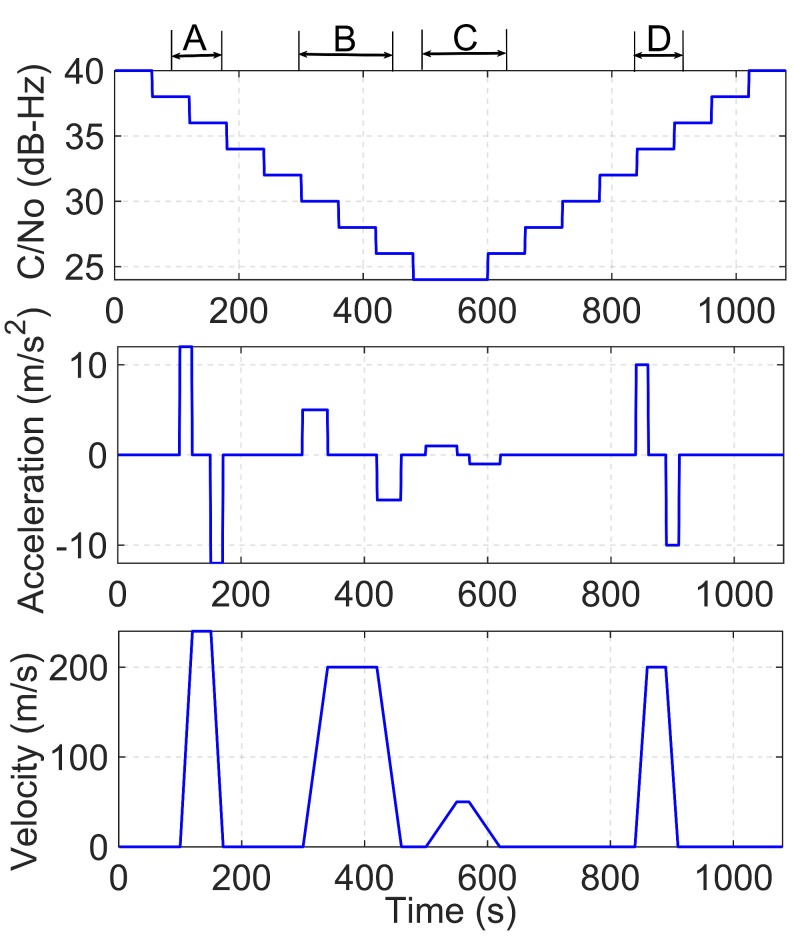
Dynamic weak signals used for the simulation.

To test the performance of the proposed algorithm under low dynamic weak signal conditions, a low dynamic situation is designed with attenuation for the signal simulation. Figure 8 shows the simulated weak dynamic signal. Regarding the signal strength, the signal started at 40 dB-Hz and descended by 2 dB every minute. The minimum power reached 24 dB-Hz at 540 s and was maintained at this level for 60 s. Finally, a recovery period with 2 dB per minute was sustained until it was resumed to the original value. For the dynamic scene simulation, the middle and bottom panels of the figure show the acceleration and velocity, respectively, of the receiver. The figure shows that four dynamic periods are simulated with different accelerations of 12 ${m/s}^{2}$, 5 ${m/s}^{2}$, 1 ${m/s}^{2}$, and 10 ${m/s}^{2}$. In each period, the receiver first accelerated with a constant acceleration, after which it moved with constant speed for tens of seconds, and finally, it slowed down with a negative acceleration until it stopped.

The IF of the simulated signal is 1.42 MHz, the sampling rate is 10 MHz, and the signal resolution is 4-bit. For comparison, two different algorithms are designed to evaluate the dynamic weak signals. The settings of CIT, $B_{nPLL}$ and $B_{nFLL}$, are the same as those of the algorithms, 2-stage conv1 and 2-stage KF1, described in Table 2. 

For two-stage KF1, the initial parameters in the fine tracking are set as follows:

(20) $$P_{0}^{-}=\mathrm{diag}[(2\pi {)}^{2}(2\pi \times 500{)}^{2}10]$$

The initialization of Q and R depends on (13) and (16), where $q_{a}$ is $0.05\left(m^{2}/s^{6}\right)/\mathrm{Hz}$ and $c/n_{0}=10^{\frac{40}{10}}$. 

**Figure 9:**
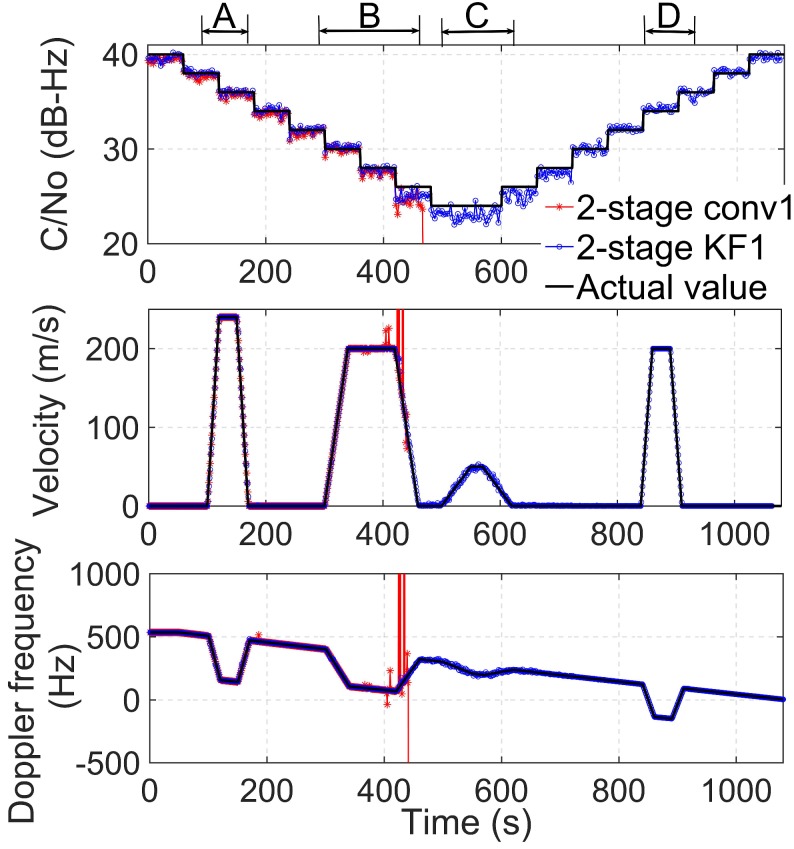
Tracking results for PRN 19 satellite signals under the dynamic weak signal condition. Top: $C/N_{0}$ estimates. Middle: velocity estimates. Bottom: Doppler frequency estimates.

The top panel of Figure 9 shows the estimated $C/N_{0}$ with the two tracking algorithms. The algorithm, 2-stage conv1, can follow the actual signal strength only when the signal power is high. When $C/N_{0}$ reaches 26 dB-Hz, the $C/N_{0}$ estimation exhibits a large error, and the receiver loses the lock at about t = 460 s. In contrast, 2-stage KF1 can still lock the signal even under the harsher environment, such as the dynamic period C, and the $C/N_{0}$ of the signal can be accurately estimated when the signal power is recovered to the high level.

The middle panel of Figure 9 shows the velocity estimated by the receiver. In general, the velocity of the receiver should be computed by more than four satellites. During the dynamic period, A, both algorithms can estimate the velocity correctly when the acceleration is 12 ${m/s}^{2}$ and when the $C/N_{0}$ is between 38 dB-Hz and 36 dB-Hz. In the dynamic period, B, the acceleration is 5 ${m/s}^{2}$ and the $C/N_{0}$ is between 30 dB-Hz and 26 dB-Hz. The algorithm 2-stage KF1 can estimate the speed correctly during the whole period, B. In contrast, the algorithm 2-stage conv1 loses the lock at about 424 s when $C/N_{0}$ is 26 dB-Hz. During the dynamic period, C, the $C/N_{0}$ is as low as 24 dB-Hz, and the acceleration is 1 ${m/s}^{2}$. The algorithm 2-stage conv1 cannot track the weak and dynamic signal after the period, B, while 2-stage KF1 can reliably maintain the lock with more than four satellites and compute the receiver velocity correctly. This observation indicates that the algorithm, 2-stage KF1, can achieve a better performance under dynamic weak signal conditions due to its variable gain in the fine tracking stage. In the dynamic period, D, $C/N_{0}$ recovers to 34 dB-Hz, and the acceleration is 10 ${m/s}^{2}$. The receiver employed with 2-stage conv1 is unable to recover to track the signal even though the signal power is recovered to a higher level. With the algorithm 2-stage KF1, the receiver can endure this variable environment and estimate the speed reliably. This indicates that the proposed algorithm outperforms the conventional two-stage tracking algorithm under variable weak and dynamic conditions. 

The bottom panel of Figure 9 demonstrates the Doppler frequency estimates of the two algorithms. The Doppler frequency is proportional to the LOS velocity. When the receiver is static, the Doppler rate changes slightly. Note that the Doppler frequency linearly changes when the receiver is accelerating. For the algorithm, 2-stage KF1, the estimated Doppler frequency is consistent with the actual values, while the estimates from the 2-stage conv1 method deviate from the actual values at approximately 410 s and lose the lock at approximately 424 s, which is consistent with the results of the middle panel of Figure 9. 

In summary, under low dynamic weak signal conditions, the proposed method shows a superior performance and strong robustness relative to the conventional two-stage tracking approach. This dynamic tracking level of the proposed method is sufficient for pedestrians and low-speed vehicles in urban canyons. 

## 6. Conclusions

A KF-based two-stage tracking method was proposed, which implemented a coarse-to-fine tracking method to improve the convergence speed and the tracking sensitivity. The coarse tracking stage reduced the large frequency error of the acquisition results for the KF-based fine tracking initialization, thereby achieving faster convergence. The fine tracking stage adopts the KF-based tracking, which can self-adjust KF gains to adapt to the challenging environments. 

Simulation results demonstrated that the proposed algorithm can improve tracking sensitivity by 8–10 dB compared with the conventional 2-stage tracking methods under static conditions. Moreover, compared with the standard KF-based tracking method, the results validate that the proposed method has a shorter convergence time. In addition, under low dynamic weak signal conditions, the proposed method was more robust than the conventional tracking algorithm. 

The proposed algorithm shows better tracking performance in the static and dynamic with pedestrian and low-speed vehicle conditions. This superiority can improve the receiver’s sensitivity and enhance the real-time performance, which is very important for positioning and navigation. However, the tracking performance of the proposed method under high dynamic conditions, such as with aircraft and missiles, is unknown. Therefore, the performance of this method under high dynamic situations should be studied in the future. On the other hand, the performance of the 2-stage KF-based tracking highly relies on the knowledge of noise statistics. However, the noise covariance is fixed during the KF iteration, which may not reflect the real variable noise statistics. Therefore, the adaptive Kalman filter can be employed in the tracking loop to further improve the tracking performance under more challenging environments.

## Figures and Tables

**Table 1:** Discriminators that are used in the FLL-assisted-PLL carrier tracking loop.

Type of Loop	Discriminator Algorithm
Second-order FLL	$\frac{\left(cross)\cdot \mathrm{sign}(dot\right)}{\sqrt{{dot}^{2}+{cross}^{2}}}$ where $cross=I_{p}(k-1)Q_{p}(k)-Q_{p}(k-1)I_{p}(k)$, $dot=I_{p}(k-1)I_{p}(k)+Q_{p}(k-1)Q_{p}(k)$, and sign(∗) denotes the sign function.
Third-order PLL	$\mathrm{ATAN}\left(\frac{Q_{P}(k)}{I_{P}(k)}\right)$ where ATAN(∗) denotes the two-quadrant arctangent.

**Table 2:** Tracking algorithms that are considered in the assessment.

Algorithms	Tracking Stage				
	Coarse Tracking			Fine Tracking	
	$B_{nPLL}(\mathrm{Hz})$	$B_{nFLL}(\mathrm{Hz})$	CIT (ms)	$B_{nPLL}$ (Hz)	CIT (ms)
2-stage conv1	15	10	4	15	4
2-stage conv2	5	10	10	5	20
2-stage KF1	15	10	4	Not applicable	4
2-stage KF2	5	10	10	Not applicable	20
